# Changes in cerebral glucose metabolism caused by morphologic features of prodromal idiopathic normal pressure hydrocephalus

**DOI:** 10.1186/s13550-019-0573-y

**Published:** 2019-12-16

**Authors:** Koichi Miyazaki, Kohei Hanaoka, Hayato Kaida, Yasutaka Chiba, Kazunari Ishii

**Affiliations:** 1 Department of Radiology, Kindai University Faculty of Medicine, Osakasayama, Osaka, Japan; 2 Institute of Advanced Clinical Medicine, Kindai University, Osakasayama, Osaka, Japan; 3 Clinical Research Center, Kindai University Hospital, Osakasayama, Osaka, Japan

**Keywords:** iNPH, DESH, AVIM, Preclinical morphologic features of DESH, FDG-PET/CT, Cerebral glucose metabolism

## Abstract

**Background:**

Decreased cerebral glucose metabolism has been reported in idiopathic normal pressure hydrocephalus (iNPH). However, the timing of appearance in the preclinical stage of iNPH remains unknown. Herein, we evaluated the changes in regional cerebral glucose metabolism with respect to the characteristic morphologic features of iNPH.

**Methods:**

We performed a cross-sectional study in > 2000 elderly patients who received a whole body 18F-fluorodeoxyglucose-positron emission tomography/computed tomography scanning and recruited subjects with clinical and preclinical iNPH. We included 12 subjects with iNPH, 32 subjects with asymptomatic ventriculomegaly with features of iNPH on magnetic resonance imaging (AVIM), and 33 subjects with preclinical morphologic features of DESH (PMD). We previously reported that iNPH develops in the order of PMD (asymptomatic subjects with incomplete DESH), AVIM (asymptomatic subjects with DESH), and iNPH (symptomatic subjects with DESH). We measured the median regional standardized uptake value ratio (SUVR) on 18F-fluorodeoxyglucose-positron emission tomography/computed tomography images between the three groups and compared them with background-matched normal controls in the frontal lobes, temporal lobes, medial parietal lobes, striata, and thalami.

**Results:**

In the frontal and temporal lobes, the SUVR distributions of the PMD, AVIM, and PMD groups were significantly lower than for each NC (*p* < 0.05 for all). In the medial parietal lobes, the SUVR distributions were significantly higher in PMD and AVIM groups (*p* < 0.05 for all). In the thalami and striata, the SUVR distributions were significantly lower in the iNPH group (*p* < 0.05 for all).

**Conclusions:**

Changes in brain glucose metabolism in the cortices are observed in preclinical iNPH, while metabolic decline in the basal ganglia is only detected in clinical iNPH.

## Introduction

Idiopathic normal pressure hydrocephalus (iNPH) is a syndrome that often develops in the elderly and is characterized by a classic triad of gait disturbance, mental deterioration, and urinary incontinence, accompanied by ventricular enlargement but normal cerebrospinal fluid pressure [1–3]. iNPH is an important disease in the elderly population, with a prevalence of approximately 1% [4–7]. Although dysfunctions caused by iNPH are treatable with shunt surgery, the cause and pathology of iNPH remain unclear. Understanding the pathology is important for improving selection of patients for shunt surgery, determining the optimal timing for therapeutic intervention, and developing new treatments.

With respect to the characteristic radiological features of iNPH, disproportionately enlarged subarachnoid space hydrocephalus (DESH) findings have been reported and are widely used for diagnosing iNPH or deciding on the indication of shunt surgery [2, 8], as they are predict outcomes after shunt surgeries. Iseki et al. also reported that DESH findings precede the onset of iNPH symptoms and termed the radiological feature in patients with asymptomatic DESH as asymptomatic ventriculomegaly with features of iNPH on magnetic resonance imaging (AVIM) [4]. AVIM is considered a preclinical stage of iNPH because of the observed transition from AVIM to iNPH.

Interestingly, Takaya et al. reported a global decrease in cerebral blood flow in iNPH patients in both the preclinical and clinical stages using single-photon emission computed tomography (SPECT) [9]. AVIM, a morphologic change, and reduced cerebral blood flow and physiological changes imply that iNPH patients show dynamic changes in the brain, even at the preclinical stage. However, at present there is insufficient information to confirm these findings. To our knowledge, only three studies have evaluated the cerebral metabolic rate of iNPH, and they reported decreased cerebral glucose metabolism using 18F-fluorodeoxyglucose-positron emission tomography/computed tomography (FDG-PET/CT) in clinical iNPH patients [10–12]. However, the stage at which the regional metabolic changes occur remains unknown and may be an important contributor to brain pathology. Therefore, we focused on the regional changes in cerebral glucose metabolism during the progression of iNPH.

Recently, we performed a cross-sectional study on >2000 elderly people who underwent a whole body FDG-PET/CT scanning and reported prevalence and morphology of cases with both preclinical and clinical iNPH [13]. In the present study, we evaluated and compared the regional cerebral FDG uptake between preclinical and clinical iNPH patients from that study with normal controls (NC) matched for each group, to elucidate the progression of changes in cerebral glucose metabolism, particularly in the preclinical stage of iNPH, and further understand the pathological mechanisms of iNPH.

## Methods

### Subjects

We retrospectively reviewed the medical records for brain images and clinical features of all 2196 patients, who are > 50 years of age and who underwent a whole body FDG-PET/CT scanning at Kindai University Hospital between May 1, 2017 and April 31, 2018 [13]. There were 79 cases with preclinical or clinical iNPH from this cohort (based on brain morphologic features of iNPH), and they were enrolled in the present study. However, two cases with clinical iNPH already had received shunt surgery prior to FDG-PET/CT scanning and were excluded from our analysis. Thus, we analyzed the FDG-PET/CT data from the remaining 77 cases with preclinical or clinical iNPH. Of these 77 cases, 92.2% had malignancy, malignant lymphoma, lung cancer, or gastric cancer, because the main purpose of the FDG-PET/CT was for cancer staging. For the NC group, a total of 89 cases were randomly selected from the same cohort of 2196 subjects, for which age, gender, and presence or absence of malignancy were adjusted to cases with preclinical and clinical iNPH.

### FDG-PET/CT scanning

After fasting for at least 4 h, each patient received intravenous 3.0 MBq/kg FDG infusion for > 2 min. After an uptake phase of 60 min, PET/CT imaging was performed on a Discovery PET/CT 710 (GE Healthcare UK Ltd., Buckinghamshire, England). CT images were acquired in helical CT mode. Helical CT acquisition was performed using a tube voltage of 120 kVp, automated tube current with a noise index of 23, rotation time of 0.5 s, detector configuration of 16 × 1.25 mm, pitch factor of 1.375 for helical CT, slice thickness of 3.75 mm, and a display field of view of 500 mm. CT data were used for attenuation correction. All PET/CT data were acquired in three-dimensional time-of-flight mode. The acquisition times per bed position was 2 min.

### Classification of clinical and preclinical iNPH

A total of 77 cases with preclinical or clinical iNPH were selected from our cohort, as described above. Case selection was performed according to DESH findings. Cases with DESH findings or incomplete DESH-like findings were adopted. The 77 cases were also classified into three groups depending on radiological features on brain CT image from PET/CT data and clinical symptoms of the iNPH triad from the patient’s medical records. The three groups were preclinical morphologic features of DESH (PMD), AVIM, and iNPH. PMD was defined as an asymptomatic subject with incomplete DESH-like findings, AVIM defined as an asymptomatic subject with complete DESH findings and iNPH as a symptomatic subject with complete DESH findings. We considered that iNPH develops in the order of PMD, AVIM, and iNPH, as reported [13]. We previously hypothesized that the progression of iNPH could be determined by PMD characteristics (i.e., younger age than AVIM and iNPH, incomplete DESH-like findings, and asymptomatic). Furthermore, we found six cases showing deformation of the brain that progressed from incomplete DESH-like findings to complete DESH findings over years. For classification of PMD, AVIM, and iNPH, the radiological features were judged by a neurosurgeon and neuroradiologist without knowledge of the clinical symptoms, and discordant cases were determined by another radiologist to make a consensus agreement between the three raters. The kappa coefficient of this classification was 0.823. A detailed description of this classification was previously reported [13].

**Fig. 1:**
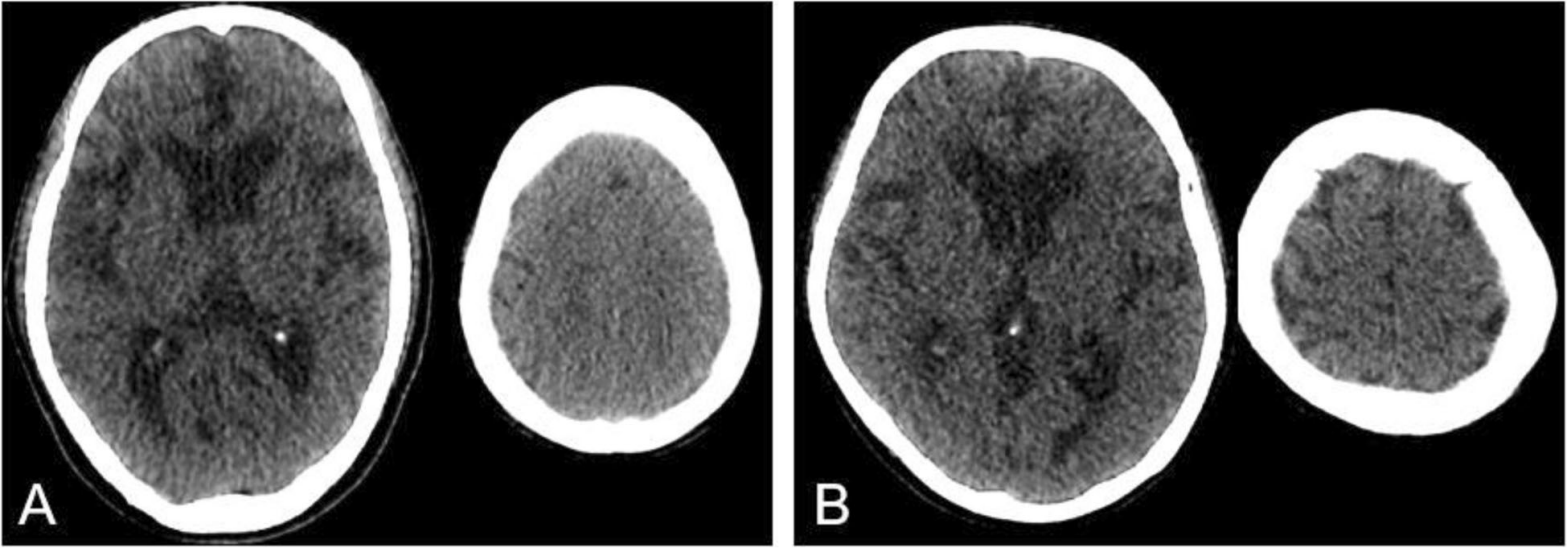
Features of disproportionately enlarged subarachnoid space hydrocephalus (DESH) and preclinical morphologic features of DESH (PMD). **a** Brain computed tomography (CT) images of a 74-year-old male subject with DESH. Evans index = 0.359, visual rating scale for tightness of the medial subarachnoid spaces and with/without tight high convexity sulci (TMC) = 3, and visual rating scale for enlarged sylvian fissures (ESF) = 2. **b** Brain CT images of an 85-year-old male subject with PMD. Evans index = 0.346, visual rating scale for TMC = 1, and the visual rating scale for ESF = 1

In brief, DESH findings consist of three components, including ventriculomegaly, tightness of the medial subarachnoid spaces and with/without tight high convexity sulci (TMC), and an enlarged sylvian fissures (ESF) [2, 8] (Fig. 1 a). We evaluated the three components independently using the Evans index for ventriculomegaly and Narita’s visual rating scale [14] for TMC and ESF. We adopted an Evans index cutoff of 0.3 to define ventriculomegaly according to the international and Japanese guidelines for iNPH [2, 15]. Narita’s visual rating scale determines the TMC and ESF of iNPH in four stages (0-3). We defined 2 or 3 as positive feature of DESH, while 1 was considered as equivocal. Cases with ventriculomegaly (Evans index > 0.3) and positive TMC and ESF (Narita’s visual rating scale of 2 or 3) were defined as DESH. DESH patients were further classified into AVIM and iNPH according to their symptoms; asymptomatic cases with DESH findings were diagnosed as AVIM, and DESH with at least one of the classic triad of iNPH were diagnosed as iNPH. Cases with one or two equivocal features of the three components of DESH findings (Evans index < 0.3 or Narita’s visual rating scale of 1) were classified as PMD, and all cases of PMD were asymptomatic (Fig. 1 b). The most frequent radiological feature of DESH findings in cases with PMD was TMC (84.8%). The characteristics of the enrolled and classified cases are described in Table 1. No brain metastasis was observed in the 77 PMD, AVIM, and iNPH cases.

**Table 1:** Demographic data

	NC for PMD (*n* = 33)	PMD (*n* = 33)	NC for AVIM (*n* = 32)	AVIM (*n* = 32)	NC for iNPH (*n* = 24)	iNPH (*n* = 12)
Age, mean (± SD)	72.0 ( ±7.1)	72.0 (± 7.1)	76.0 (± 5.3)	76.0 (± 5.3)	78.8 (± 6.0)	78.8 (± 6.0)
Sex, male/female	27/6	27/6	22/10	22/10	14/10	7/5
Malignancy, presence/absence	30/3	30/3	30/2	30/2	22/2	11/1
Evans index, mean (± SD)	0.271 (± 0.024)	0.315 (± 0.032)	0.276 (± 0.027)	0.339 (± 0.027)	0.277 (± 0.018)	0.349 (± 0.021)
Visual rating scale for TMC 0, dilated/1, normal/2, mildly tight/3, severely tight	2/31/0/0	0/5/9/19	1/30/1/0	0/0/11/21	1/23/0/0	0/0/5/7
Visual rating scale for ESF 0, narrowed/1, normal/2, mildly dilated /3, severely dilated	6/15/11/1	0/14/5/14	1/20/11/0	0/0/14/18	4/16/4/0	0/0/7/5

*SD* standard deviation, *TMC* tightness of the medial subarachnoid spaces and with/without tight high convexity sulci, *ESF* enlarged sylvian fissures, *NC* normal control, *PMD* preclinical morphologic features of disproportionately enlarged subarachnoid-space hydrocephalus (DESH), *AVIM* asymptomatic ventriculomegaly with features of iNPH on magnetic resonance imaging, *iNPH* idiopathic normal pressure hydrocephalus

### Image data analysis

**Fig. 2:**
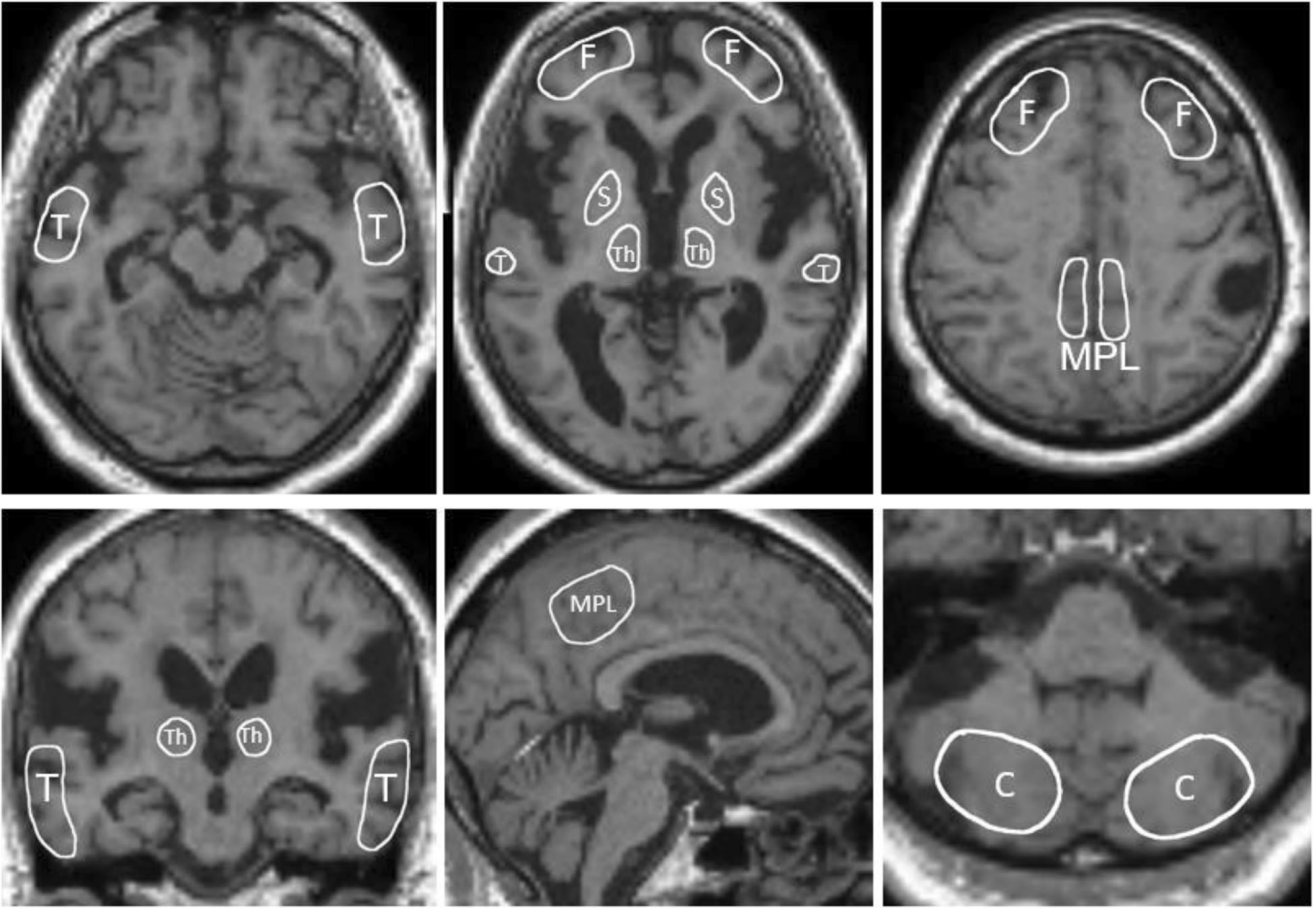
Representative slices of volumes of interest (VOIs) used to evaluate lesion-to-cerebellum standardized uptake value ratio (SUVRs). All VOIs were drawn to avoid areas of brain deformation, such as ESF, dilated ventricles, and focally dilated sulci. For the purpose of clearly showing the location of the VOI, this figure was created using magnetic resonance imaging (MRI) images of DESH. *F* frontal lobes, *T* temporal lobes, *MPL* medial parietal lobes, *S* striata, *Th* thalami, *C* cerebellum

DICOM PET data were converted to NIfTI format for processing on MRIcron software (University of South Carolina, Columbia, SC, USA). PET data were then spatially normalized and smoothed on Statistical Parametric Mapping version 12 (SPM12; Institute of Neurology, University College London, UK) implemented on MATLAB R2013b (The MathWorks Inc., Natick, Mass, USA). Volumes of interest (VOIs) were drawn for five brain regions (frontal lobes, temporal lobes, medial parietal lobes, striata, and thalami) and the cerebellum, avoiding deformation of the brain and CSF space in iNPH patients (Fig. 2). Using these VOIs, the standardized uptake value ratio (SUVR) of the five brain regions with reference to the cerebellar cortex was calculated on MRIcron for each subject.

### Statistical analysis

SUVR values are presented as medians and interquartiles. The locations of the SUVR distributions for each of the three groups were compared with each normal control group, region by region, using the Mann-Whitney U test. All statistical analyses were performed with statistical software (R v3.4.1; downloadable at https://cran.r-roject.org/bin/windows/base/old/3.4.1/). The statistical significance level was set at *p* < 0.05.

## Results

**Fig. 3:**
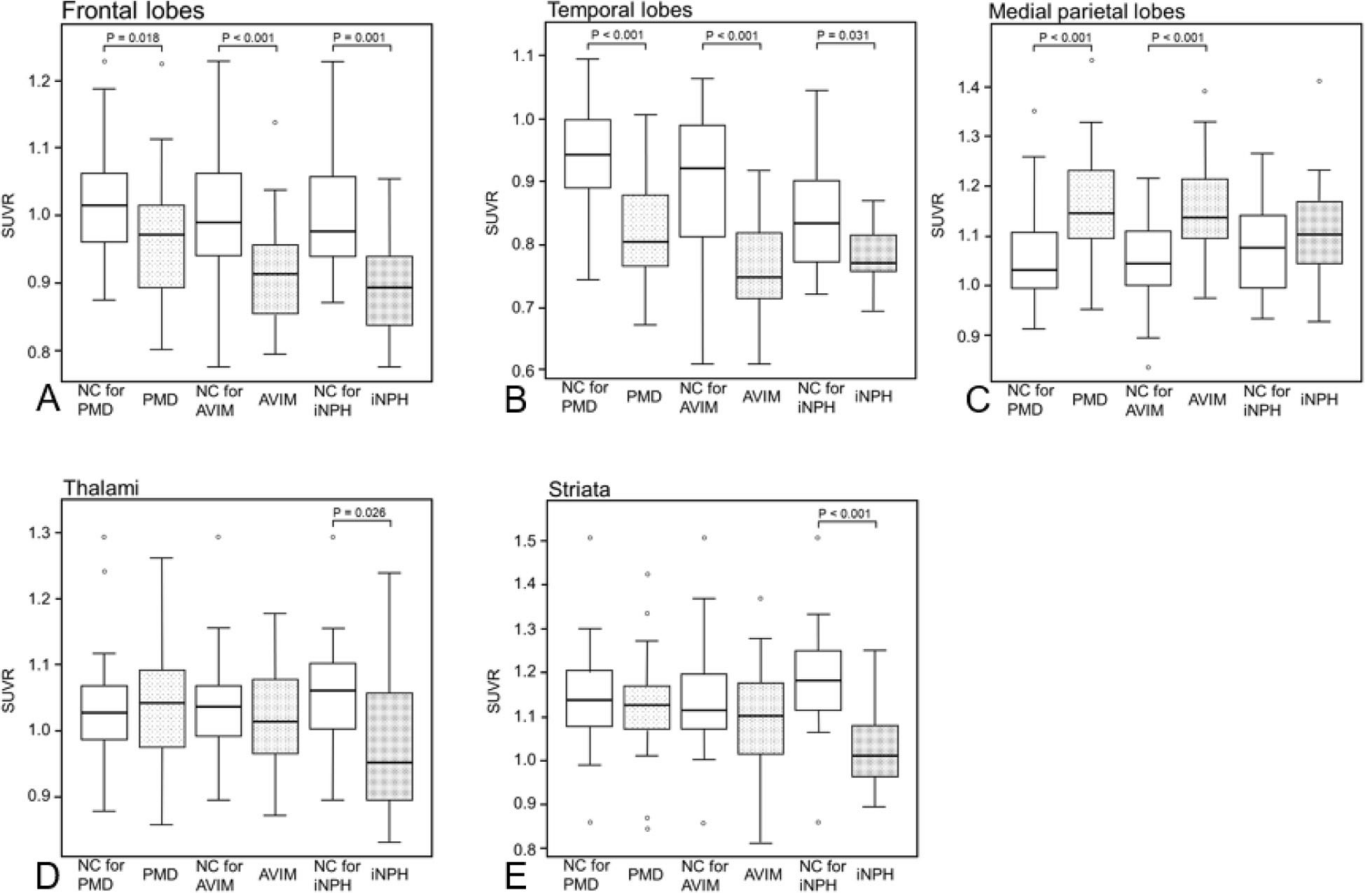
SUVRs of the five VOIs for each of the three pathological groups and their normal controls (NC). Idiopathic normal pressure hydrocephalus (iNPH) may develop in the order of PMD, asymptomatic ventriculomegaly with features of iNPH on MRI (AVIM) and iNPH. PMD: asymptomatic subjects with incomplete DESH, AVIM: asymptomatic subjects with complete DESH, iNPH: symptomatic subjects with complete DESH. All NC groups were adjusted to their corresponding pathological groups based on their background data. **a** and **b** in the frontal and the temporal lobes, all PMD, AVIM and iNPH groups showed significantly lower SUVRs than that of corresponding NC groups. **c** in the medial parietal lobes, the PMD and AVIM had significantly higher SUVRs than that of corresponding NC groups. **d** and **e** in the thalami and the striata, only the iNPH group showed a significantly lower SUVR than that of the NC group

The median and interquartile ranges of all SUVRs are shown in Table 2. In the frontal lobes and temporal lobes, the SUVR distributions for the PMD, AVIM, and iNPH groups were significantly less than those for each NC group (Table 2, Fig. 3a, b). In the medial parietal lobes, the SUVR distributions in the PMD and AVIM groups were significantly higher than those for each NC group (Table 2, Fig. 3c). In the thalami and striate, the SUVR distributions were lower in the iNPH group compared with those in the NC group (Table 2, Fig. 3d, e).

**Table 2:** standardized uptake value ratio (SUVR) values in each region

Groups	Frontal lobes	*p* value	Temporal lobes	*p* value	MPL	*p* value	Thalami	*p* value	Striata	*p* value
NC for PMD	1.013 (0.961-1.062)	0.018^a^	0.942 (0.890-0.997)	< 0.001^a^	1.030 (0.994-1.108)	< 0.001^a^	1.028 (0.988-1.069)	0.939	1.138 (1.079-1.206)	0.76
PMD	0.972 (0.892-1.015)		0.806 (0.776-0.875)		1.145 (1.094-1.230)		1.042 (0.974-1.091)		1.127 (1.071-1.171)	
NC for AVIM	0.988 (0.941-1.059)	< 0.001^a^	0.920 (0.819-0.986)	< 0.001^a^	1.045 (1.002-1.108)	<0.001^a^	1.036 (0.990-1.067)	0.475	1.118 (1.075-1.194)	0.139
AVIM	0.914 (0.853-0.957)		0.749 (0.716-0.819)		1.135 (1.093-1.212)		1.015 (0.964-1.077)		1.105 (1.019-1.179)	
NC for iNPH	0.977 (0.943-1.055)	0.001^a^	0.832 (0.774-0.896)	0.031^a^	1.078 (0.998-1.131)	0.497	1.061 (1.005-1.098)	0.026^a^	1.180 (1.119-1.250)	< 0.001^a^
iNPH	0.891 (0.840-0.929)		0.772 (0.759-0.815)		1.104 (1.056-1.164)		0.952 (0.901-1.041)		1.010 (0.966-1.074)	

*MPL*, medial parietal lobes

SUVR values are presented as median (25th and 75th percentiles of the interquartile range)

^a^*p* value < 0.05 (Mann-Whitney U test)

## Discussion

To our knowledge, this is the first report assessing the changes in regional cerebral glucose metabolism using FDG-PET/CT in the prodromal stage of iNPH. We previously reported that iNPH develops in the order of PMD, AVIM, and iNPH [13], with PMD (asymptomatic incomplete DESH) progressing to AVIM (asymptomatic complete DESH), which processes to iNPH (symptomatic complete DESH). In this study, we also evaluated cerebral glucose metabolism during the progression of iNPH.

In the cortices of the frontal lobes and the temporal lobes, all three groups showed lower SUVRs than each of the NC groups, confirming decreased cerebral glucose metabolism. In an FDG-PET/CT study, Calcagni et al. reported that brain glucose metabolism was globally decreased in iNPH cases at preshunt surgery compared with post-shunt surgery [12] and suggested that the global decrease in cerebral glucose metabolism was related to the clinical symptoms of iNPH. However, in the present study, we found a decreased metabolism in the frontal and temporal lobes before the onset of iNPH; i.e., the decrease was observed even at asymptomatic stages. This contrasts with the findings of Townley et al. who reported no hypometabolism in the frontal and temporal cortices when using partial volume correction on MRI images [10]. Nevertheless, the figures in that study actually showed decreases in cerebral metabolism in the frontal and temporal cortices, even below the threshold, while they only assessed seven cases. In the present study, because of the lack of MRI images, we were unable to perform the same analysis method as that by Townley et al. Furthermore, the influence of sulci deformation in the VOIs is unknown. However, we placed the VOIs in the frontal and temporal lobes in a position where there was little deformation of the DESH brains. Thus, we consider that cerebral glucose metabolism was decreased in those cortices of the iNPH brain. Further studies of more cases are required to confirm our findings.

We also found a significant increase in cerebral metabolism in the medial parietal lobes in the PMD and AVIM groups compared with the NC group, which was opposite to that reported by Calcangi et al. This discrepancy may be caused by the different VOIs used between the studies. For placement of the parietal VOI, Calcangi et al. used part of the lateral cortices. However, we used the medial cortices to place the parietal lobe VOI. Gray matter density was reported to be increased in the medial parietal cortices of iNPH patients [16], which may contribute to our findings of elevated SUVR in PMD and AVIM. Based on our findings of a significant increase in SUVRs in PMD and AVIM patients, but not in iNPH patients, we suggest that cerebral glucose metabolism in the medial parietal area also tends to decline with progression of hydrocephalus.

In the basal ganglia (the striata and thalami), there was a significant decrease in the SUVRs only in the iNPH group. These data are consistent with a previous report that striatal glucose metabolism in iNPH is reduced compared with normal healthy controls [10]. Interestingly, the change in regional cerebral glucose metabolism proceeded the onset of iNPH, whereas in the basal ganglia, SUVRs decreased late in the disease (i.e., in developed iNPH). Thus, glucose metabolism in the basal ganglia is decreased when hydrocephalus become symptomatic, suggesting that hypothesis that the basal ganglia may contribute to the symptoms of iNPH, although further studies are required to confirm these findings.

The present study has several limitations. First, the three pathological groups (PMD, AVIM, and iNPH) were obtained from our previous cross-sectional study. Thus, we were unable to clearly define whether iNPH actually developed in the order of PMD, AVIM, and iNPH. However, the morphologic features of iNPH, the presence or absence of symptoms, and the progression of several cases with serial MRI support our hypothesis of this progression [13]. Future prospective longitudinal studies with long-term follow-up are required to show this development of iNPH. Second, because MRI images were not obtained, we were unable to perform a partial volume correction to eliminate the influence of a deformed subarachnoid space of the iNPH brain, as previously reported [10]. Therefore, we were unable to totally exclude the potential bias from the deformation, especially at the cortices. Nevertheless, for every case, we confirmed that the rough deformations (enlarged ventricles, enlarged sylvian fissures, and focally dilated sulci) were not included with the VOIs. Third, the majority of subjects in our study had cancer, and we cannot exclude a potential effect of malignancy on cerebral glucose metabolism. Indeed, several FDG-PET/CT reports have shown alterations in cerebral glucose metabolism in patients with malignancy [17, 18]. For this reason, we created three normal control groups for each pathological group to adjust the malignancy rate and other backgrounds and remove the potential confounding effects of cancer. Finally, the VOIs were drawn by the first author, and it is difficult to generalize current results. Thus, the use of software that automatically measures and compares SUVRs may be preferable, although we found that the intense deformations in the brain of iNPH patients can affect the results using such software. As such, we used manual VOIs to avoid the deformed brain regions.

## Conclusions

Glucose metabolism in the cortices was altered even in the preclinical stages of iNPH. By contrast, decreased glucose metabolism in the basal ganglia was only observed in clinical iNPH, but not in the asymptomatic preclinical stage. These novel findings provide further information on the potential mechanisms and progression of iNPH.

## Data Availability

The datasets and images used during the current study are available from the corresponding author on reasonable request.

## Abbreviations

iNPH: Idiopathic normal pressure hydrocephalus
DESH: Disproportionately enlarged subarachnoid-space hydrocephalus
AVIM: Asymptomatic ventriculomegaly with features of iNPH on magnetic resonance imaging
PMD: Preclinical morphologic features of DESH
SUVR: Standardized uptake value ratio
SPECT: Single-photon emission computed tomography
FDG-PET/CT: 18F-fluorodeoxyglucose-positron emission tomography/computed tomography
TMC: Tightness of the medial subarachnoid spaces and with/without tight high convexity sulci
ESF: Enlarged sylvian fissures
NC: Normal controls
VOIs: Volumes of interest
